# How to make the best use of the workload indicators of staffing needs method in determining the proportion of time spent in each of the workload components and its implication in decision making: the experience of the Sultanate of Oman

**DOI:** 10.1186/s12960-021-00656-2

**Published:** 2022-01-28

**Authors:** Nazar Mohamed, Nahida Al-Lawati

**Affiliations:** 1 Adviser Human Resources Planning, Ministry of Health, Muscat, Sultanate of Oman; 2 Head of Policy and Health Systems Unit, Ministry of Health, Muscat, Sultanate of Oman

**Keywords:** Workload indicators of staffing needs, Primary health care institution, Workload pressure, Proportion of time

## Abstract

**Background:**

The Ministry of Health in the Sultanate of Oman decided to have better distribution of the health workforce among all health facilities through evidenced-based staffing norms. Four directorates worked together to develop the staffing norms through making use of the workload indicators of staffing needs (WISN) method. The aim of this study is to describe the process of applying the WISN method in Primary Health Care institutions and how to make the best use of method in determining the proportion of time spent in each of the workload components and its implication in decision making.

**Methods:**

The WISN was applied for five priority categories, namely, doctors, nurses, pharmacists, laboratory technicians, and radiology technicians at PHC institutions. The WISN ratio has been translated into workload pressure as a percentage through applying the formula [workload pressure as % (in case of shortage) = (1 − WISN ratio) × 100%]. While the proportion of time spent in each of the workload components was calculated through making use of the category allowance standard, the individual allowance standard to determine the time spent in support and additional activities. The sum is subtracted from 100% to give the time spent in the health service activities.

**Results:**

Determining the workload pressure as a percent and its interpretation is based on the fact that one cadre or as a group can bear up to 10% of extra workload. Thus, managers can undertake sensible short-term arrangements or decisions in redistributing the cadres among the health facilities on expectation of deploying more staff.

**Discussion:**

Careful and detailed analysis of the proportion of time spent in each of the workload components will allow to have better understanding of the context and dynamics of work.

**Conclusion:**

Decision makers and planners can undertake rational short-term decisions in redistributing the cadres among the health facilities based on the workload pressure. In addition, they can as well as easily decide on the optimal proportions of time for each staff category, and hence choose what activities and tasks to be shifted or delegated to other staff category.

## Background

The Sultanate of Oman is located in the south eastern corner of the Arabian Peninsula. Administratively, the country is divided into 11 governorates and 61 districts, locally known as (Wilayat) distributed among these governorates. The population size in 2019 was 4,617,927 of whom 42.5% were non-Omani [1].

Oman has achieved notable progress towards universal health coverage (UHC) through dramatic transformation in its health care system over a remarkably short span of time [2]. The country has being recognized internationally as one of the few countries with successful experience in health development. For instance, the World Health Organization (WHO), in its first-ever comparative analysis of health systems in 2000, ranked Oman first among 191 countries for its overall performance on the level of health [3]. In 2010, the United Nation Development Programme (UNDP) identified top mover countries relative to the starting point in 1970s regarding health development and ranked Oman first among 135 countries worldwide as the most improved nation during the preceding 40 years [4].

Concerning for the health workforce, the density of doctors and nurses per population in Oman has increased remarkably. As for doctors, it reached 20.8 per 10,000 population in 2019 compared to 5.1 in 1980, while for the nurses, it reached 44.0 per 10,000 population compared to 10.8 in 1980 [1]. The country continued to develop its educational and training infrastructure and began to produce as much health professionals as possible, to meet health care demands and achieve workforce self-reliance.

Different initiatives with a beneficial impact on the workforce development were introduced in the past decades to foster the nationalization policy and retention of the health workforce such as the regionalization of health professions’ training institutes, active collaboration with universities and overseas specialty boards, qualitative improvement of the education system and the development of a strong continuing professional development system [5]. It is worth to note that the workforce management system treats both males and females health professions on equal footing and there is no discrimination in terms of recruitment, deployment, remuneration, and professional development.

Improving health workers’ performance and productivity is vital for better health service provision in the country. Thus, the Ministry of Health (MoH) in Oman recognized that having the best talented, motivated and competent health care professionals with the right number and skill-mix are very critical in addressing the system challenges, tackling the burden of diseases and sustaining UHC through provision of quality health care services [6]. This vision is in alignment with the Health Workforce 2030 Global Strategy [7] which has reaffirmed that health workforce will be critical to achieving health and wider sustainable development goals (SDGs). The health targets under consideration in the SDGs include a renewed focus on equity and UHC [7]. This can be attained through substantive and strategic investments in health workforce for both males and females.

Improving health services coverage and health outcomes are much dependent on the availability, accessibility, acceptability and quality human resources for health [7, 8].

In the 9th 5-year health development plan (2016–2020), the MoH decided to have better distribution of the health workforce among all health facilities (health centers and hospitals) through evidenced-based staffing norms [9, 10]. Hence, four directorates, namely, Directorate General of Planning and Studies, the Directorate General of Primary Health Care (PHC), the Directorate General of Nursing Affairs, and the Directorate General of Medical Supplies worked together to develop the staffing norms through making use of the workload indicators of staffing needs (WISN) method, developed by the World Health Organization WHO).

The plan to achieve the desired outcome was divided into phases. The first phase developed the national norms based on national averages and assumptions without applying the WISN at individual health facilities, and the results were published in 2018 [10]. The second phase was to apply the WISN method at health facility level.

Hence, the aim of this paper is to describe the process of applying the WISN method in verifying and adjusting the national norms for PHC facilities and how to make the best use of method in determining the proportion of time spent in each of the workload components and its implication in decision making.

## Methods

The WISN steps were followed in applying the tool at governorate and health facility level, i.e., establishing the structures (steering committee, technical team and experts group), determining priority cadres and health facility types, estimating the available working time (AWT), defining the workload components, setting the activity standards, establishing the standard workloads, calculating the category allowance standard (CAS) and category allowance factor (CAF), individual allowance standard (IAS) and individual allowance factor (IAF), computing the staffing requirements based on WISN, and last analyzing and interpreting WISN results. The WHO WISN user manual describes these steps in detail. [11].

The workload components are the tasks performed by each staff category and are divided into three types: health service activity (A) which are activities performed by all members of the staff category and measured by regular collection of statistics; support activity (B) which are performed by all members of the staff category but statistics are not regularly collected; and additional activities (C) which are performed only by certain members of staff category [11].

The study was carried from 2017 to 2019, and the national norms were obtained after an extensive piloting and testing exercise in the 11 governorates which covered 128 PHC institutions (represent 54% out of the total 238 PHC institutions in the MoH). The aggregation of data from those PHC institutions was used to develop the norms and standards.

The WISN was applied for five priority categories, namely, doctors, nurses, pharmacists, laboratory technicians, and radiology technicians at PHC institutions. Six training workshops were conducted on the WISN method and 218 participants representing those categories attended those workshops.

Data used to calculate the AWT, activity standards, and annual workload were obtained from the unified electronic health information system (named ALSHIFA) and the human resources management (HRM) information system. All MoH facilities (hospitals and health centers) are linked to ALSHIFA system, whereby all activities (tasks performed by health cadres such as seeing patients, procedures, medications, investigations, admissions, etc. are recorded). It is worth to mention that the roles, responsibilities and duties of doctors, nurses, and the other cadres are standardized in all Ministry of Health PHC centers (regardless to the geographical location of the facility or the density of the population) and are linked to the unified electronic information system (ALSHIFA). While the HRM information system records all types of leaves and absenteeism.

Different means were used to verify the data and standards such as comparing the statistics of the PHC institution (monthly reports) vis-a-vis ALSHIFA system, consulting the expert groups, interview of the cadres, as well as time–motion observation. Where activities are shared between two cadres (e.g., vaccination or antenatal care between doctor and the nurse), the activity standard was divided between the two cadres according to the time spent by each cadre.

### Calculating the workload pressure

In addition to determining the staff requirements based on WISN, comparing between the current and required staff, and calculating the WISN ratio; this study shows how to translate the WISN ratio into workload pressure as a percentage in case of shortage through applying the formula [workload pressure as % (in case of shortage) = (1 − WISN ratio) × 100%].

### Calculating the proportion of time spent in each of the workload components

This study also shows how to calculate the proportion of time spent in each of the workload components. To determine the proportion of time, we used the CAS which is percentage of working time spent in performing the support activities (B), and then calculated the percentage of time spent in the additional/individual activities (C) through applying the following formula [IAS/(AWT × total no. of current staff) × 100%]. By adding the percentage of time spent in B and C activities, the remaining will be the percentage of time spent in A activities, i.e., this can be calculated as follows: [100 – (% of time allocated for B + % of time allocated for C)].

## Results

Although the WISN method has been applied in all the PHC institutions under the study, the results displayed hereunder are giving examples on the importance of measuring the workload pressure and the proportion of time distributed among the workload components, and their implication on planning and decision making.

### Verifying and adjusting the national norms for PHC facilities

Following the application of the WISN method in the PHC institutions (128), data were aggregated, verified, and standardized; and the national norms were developed for the five priority categories. The AWT, CAF, and IAF were standards, as shown in Table 1. The variations in the AWT were due to the difference in the annual leave allocated for each cadre. As for the health service activities (A), the activity standards were unified for all the workload components (for the five priority staff categories).

**Table 1:** Standardized norms for the five priority categories

Staff category	AWT (hours)	CAF	IAF
Doctors	1316	1.2	0.5
Nurses	1323	1.3	1
Pharmacists	1358	1.3	0.5
Lab technicians	1379	1.3	0.1
Radiographers	1323	1.2	0.2

### The workload pressure

Table 2 shows the aggregate results of the WISN in Muscat governorate for doctors and nurses (a total of 26 PHC institutions). There was shortage in both cadres (− 68 and − 26 for doctors and nurses, respectively). The overall WISN ratios (for the whole governorate) were 0.8 and 0.9, and the workload pressure was 20% and 10% for doctors and nurses, respectively. However, there were differences in the ratio between the PHC institutes (> 1, 1, and < 1).

**Table 2:** Aggregated results of the WISN for doctors and nurses, Muscat governorate

Muscat governorate	WISN	Current	Difference	WISN Ratio	Workload pressure (%)
Doctors	338	270	− 68	0.8	20%
Nurses	439	413	− 26	0.9	10%

To determine the severity of the shortage and whether the cadre can cope with the extra workload, we analyzed (in depth) the formula of rounding the fractional results of the required staff in the WHO WISN user manual, as shown in Table 3.

**Table 3:** How to round the fractional results

Fraction	Rounding	Fraction	Rounding
1.0–1.1	Rounded down to 1	> 1.1–1.9	Rounded up to 2
2.0–2.2	Rounded down to 2	> 2.2–2.9	Rounded up to 3
3.0–3.3	Rounded down to 3	> 3.3–3.9	Rounded up to 4
4.0–4.4	Rounded down to 4	> 4.4–4.9	Rounded up to 5
5.0–5.5	Rounded down to 5	> 5.5–5.9	Rounded up to 6

We determined from the formula of rounding the fractions that 0.1 in staff means as if you need extra 0.1 staff to cope with the workload (which is equivalent to 10%). However, the formula recommended this fraction (0.1) to be rounded down. When the fraction is more than 0.1, the workload is more than 10% and consequently the fraction is rounded up. This is applied to all the fractions showed in table (3), i.e., 2.2 staff means the 0.2 is 10% extra workload for each of the two, 3.3 means the 0.3 is 10% extra workload for each of the 3. Thus, the fraction is rounded down and no need for an additional staff. However, if the fraction is > 2.2–2.9, > 3.3–3.9, > 4.4–4.9, > 5.5–5.9; it means that workload is more than 10% on average, and you need additional staff to bear the extra workload and thus the fraction is rounded up.

We concluded that one staff can bear up to 10% of workload, and if it is more than 10% an additional staff is needed. This conclusion can be applied to individual PHC institutions or as aggregate. Therefore, concerning the WISN results of Muscat governorate table (2), the current nursing staff can cope with the extra workload pressure (10%) in spite of the shortage, while additional doctors are needed to cope with the workload pressure (20%).

### Proportion of time spent in each of the workload components

**Fig. 1:**
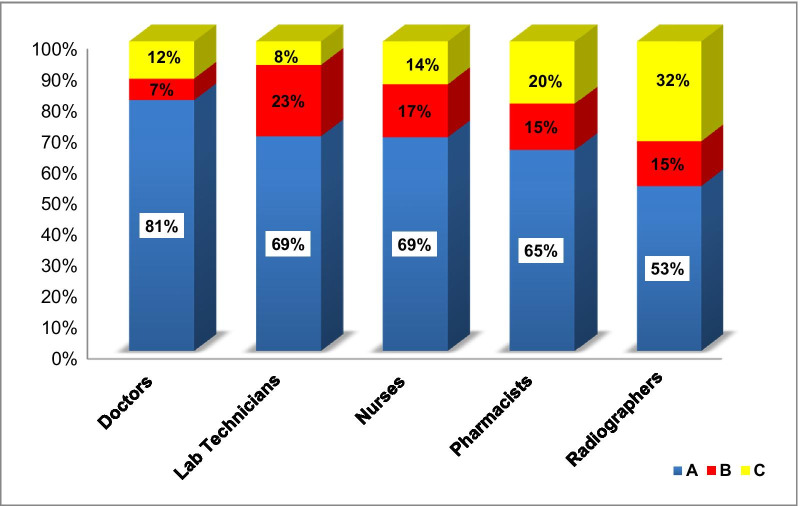
Proportion of time spent for each of the five priority staff categories, Dhofar governorate

We have calculated the proportion of time (as a percentage) spent in each of the workload components; health service activities (A), support activities (B), and additional activities (C). Figure 1 shows the calculations for each of the five priority staff categories in Dhofar governorate. When further analyzing the data for lab technicians, it was evident that they spent more time in (B) activities (23%) due to calibration of the devices. On the other hand, radiographers spent more time in (C) activities (32%), because the machines were old and a significant time was spent in maintenance.

**Fig. 2:**
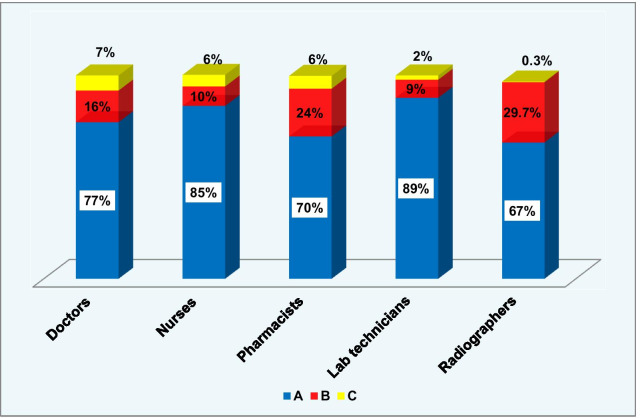
Proportion of time spent for each of the five priority staff categories, South Al-Batinah governorate

Figure 2 shows the calculations for each of the five priority staff categories in South Al-Batinah governorate. When further analyzing the data for radiographers, it showed that they spent considerable time in (B) activities (29.7%), because the X-ray machine was not advanced in technology and they perform more manual work in preparing the films. While pharmacists spent 24% of their time in (B) activities (arranging the drug stores).

**Fig. 3:**
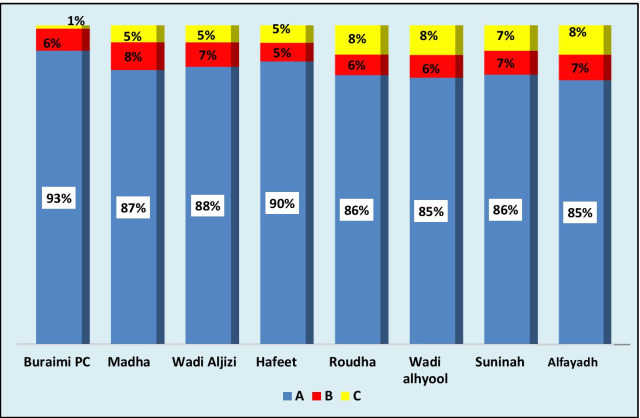
Proportion of time spent by doctors at PHC institutions, Alburaimi governorate

Figure 3 shows the calculations for doctors at PHC institutions in Alburaimi governorate. The data showed similarity in proportion of time spent by doctors in the three workload components in all the PHC institutions. However, the proportion of time spent in (B) activities was less than the national average.

**Fig. 4:**
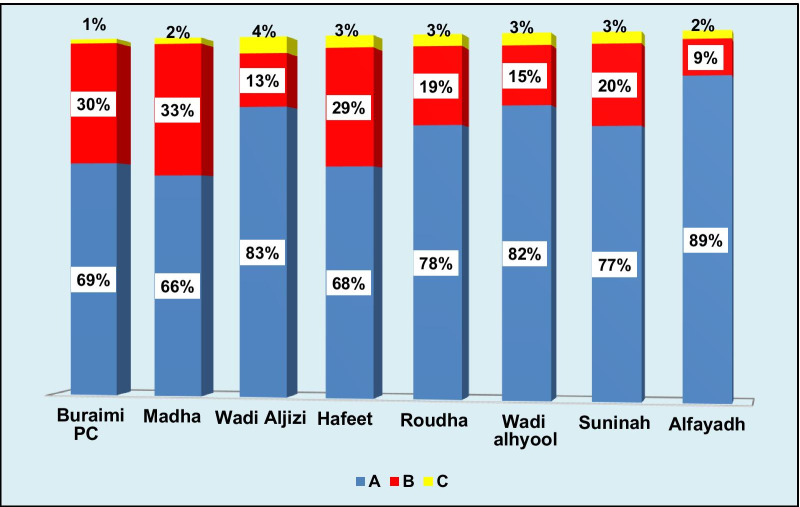
Proportion of time spent by nurses at PHC institutions, Alburaimi governorate

On the contrary, the same pattern was not realized for the nurses in the same PHC institutions in the same governorate, as shown in Fig. 4. There was variation in the proportion of time spent in (B) activities ranging between 9 and 33%. When further analyzing the results, it was observed that some PHC institutions assigned more tasks to nurses which were classified as support activities (B).

## Discussion

Establishing the staffing norms is of paramount importance. Many countries managed to do so through using the WISN method (which is easier to comprehend and use) either at national or district levels (big WISN as indicated in the WHO WISN user manual) such as Bangladesh, Burkina Faso, Ghana, Greek, Kenya, Namibia, South Africa, Uganda, and others [12–19].

Although two types of results (differences and ratios) are provided by the WISN method, we managed to add other useful dimensions (the workload pressure as a percentage and the proportion of time spent in each of the workload components).

Determining the workload pressure as a percent and its interpretation is based on the fact that one cadre or as a group can bear up to 10% of extra workload (as the case of Muscat governorate). Hence, managers can undertake sensible short-term arrangements or decisions in redistributing the cadres among the health facilities on expectation of deploying more staff. However, if the workload pressure exceeds 10%, then converging the shortage by adding staff or implementing measures to reduce the workload will be the best options. Otherwise, the negative consequences of the shortage will become apparent such as compromising the quality of the services, fatigue, burnout, lack of motivation, medical errors, violence, quitting, etc. [12, 15, 20].

Calculating the proportion of time spent in each of the workload components is very useful in knowing how the work is distributed within the same staff category or among different categories. We realized that most of the workload components under group (A) activities have direct contribution to the desired outcomes (e.g., seeing patients, vaccination, antenatal and postnatal care, nursing procedures, dispensing drugs, laboratory investigations, X-rays, etc.). On the other hand, the activity components under (B) and (C) contribute (most of the time) indirectly to the desired outcomes (e.g., meetings, trainings, data entry, recording, supervision, preparing rosters, calibration of devices, etc.). Thus dedicating more time in performing the activities under (A) is desirable.

While calculating the proportion of time spent in (B) activities, we made use of both the CAS as a percentage, and the CAF. It was found that 7–8% increment in the CAS will increase the CAF by 0.1, i.e., if the CAS is 17%, the CAF will be 1.2, and if the CAS is 25%, the CAF will be 1.3. The 17% and 25% represent the proportion of time spent in (B) activities. Thus, any increase in the CAS (in terms of proportion of time spent in B) will be on the expense of the proportion of time (A) activities and consequently the desired outcomes.

Careful and detailed analysis of the proportion of time spent in each of the workload components based on the ideal task analysis will allow to have better understanding of the context and dynamics of work. Decision makers and planners can easily decide on the optimal proportions of time for each staff category, and hence choose what activities and tasks to be shifted or delegated to other staff category.

## Conclusion

Decision makers and planners are responsible for managing effectively and efficiently the health workforce to ensure that the right number of healthcare workers, with the right knowledge, skill-mix, attitudes and qualifications are performing the correct tasks in the right place at the appropriate time to achieve predetermined health targets.

This study demonstrated how to make the best use of the WISN method and its usefulness in estimating staffing requirements, thus providing evidence-based solutions and recommendations for better planning. Careful and detailed analysis of the workload pressure and proportion of time spent in each of the workload components will allow to have better understanding of the context and dynamics of work.

Decision makers and planners can undertake rational short-term decisions to ensure equity in redistributing the cadres among the health facilities based on the workload pressure and task analysis. In addition, they can easily decide on the optimal proportions of time for each staff category, and hence choose what activities and tasks to be shifted or delegated to other staff category.

## Data Availability

The data sets used and/or analyzed during the current study are available from the corresponding author on reasonable request. All relevant raw data, will be freely available to any scientist wishing to use them for non-commercial purposes. All data generated or analyzed during this study are included in this published article [and its supplementary information files].

## Abbreviations

AWT: Available working time
CAF: Category allowance factor
CAS: Category allowance standard
IAF: Individual allowance factor
IAS: Individual allowance standard
MoH: Ministry of Health
PHC: Primary health care
SDGs: Sustainable development goals
UHC: Universal health coverage
WHO: World Health Organization
WISN: Workload indicators of staffing needs
